# Supplementary material for: Surface Modification of UiO-66 on Hollow Fibre Membrane for Membrane Distillation
Source: Membranes (Basel). 2023 Feb 21;13(3):253. doi: 10.3390/membranes13030253 (PMC10055739; doi:10.3390/membranes13030253)
Supplement: Supplementary file 1 [file membranes-13-00253-s001.zip › membranes-2165707-supplementary.pdf]

Surface Modification of UiO-66 on Hollow Fibre Membrane for Membrane Distillation

Noor Fadilah Yusof [1], Amirul Afiat Raffi [1], Nur Zhatul Shima Yahaya [1], Khairul Hamimah Abas [2], Mohd Hafiz Dzarfan Othman [1], Juhana Jaafar [1] and Mukhlis A. Rahman [1,*]

[1] Advanced Membrane Technology Research Centre (AMTEC), Universiti Teknologi Malaysia, 81310 Skudai, Johor, Malaysia.
[2] Department of Control & Instrumentation Engineering, School of Electrical Engineering, Universiti Teknologi Malaysia, 81310 Skudai, Johor, Malaysia.
*Corresponding author: r-mukhlis@utm.my
Tel: +607-553-6373, Fax: +607-533-5925

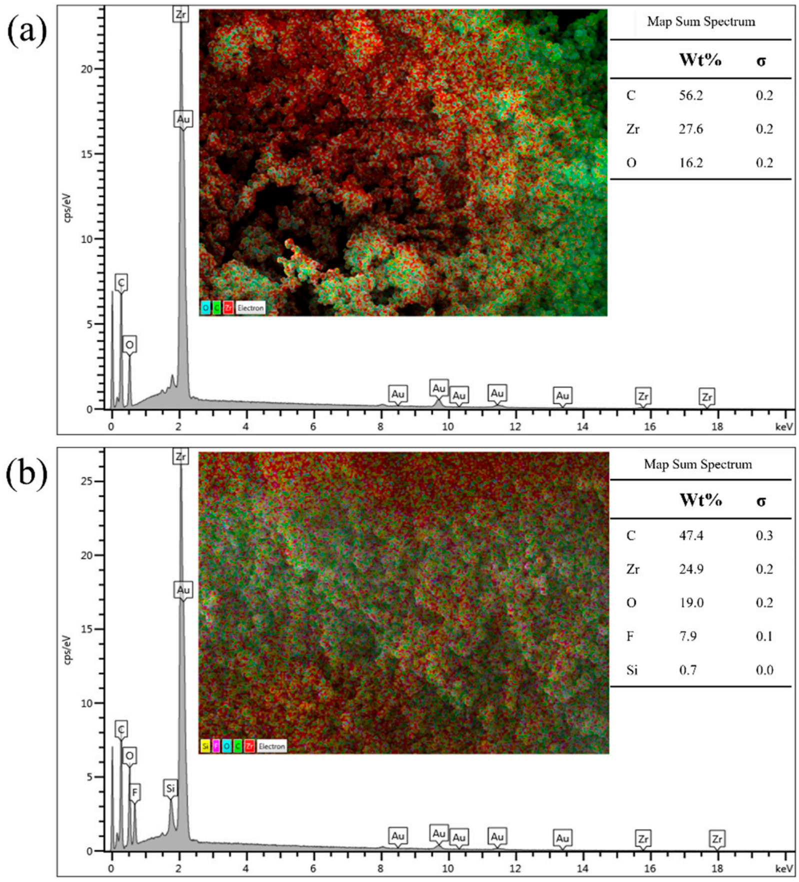

Figure S1. EDX mapping of (a) UiO-66 and (b) FAS grafted onto the UiO-66 layer

Water flux and salt rejection calculation:

Table S1. The linear regression equation of NaCl concentration

NaCl concentration (ppm)	Equation
2 - 10	y = 2.62x + 3.106
10 - 60	y = 1.976x + 15.173
20 - 100	y = 2.490x + 19.65
2000 – 10,000	y = 0.0016x + 0.37
20,000 – 100,000	y = 0.0014x + 7.29

Water flux, $J_w = \frac{V}{t \times A}$ V = water collected (L)

t = time (8h)

A = total surface area (9.28x10^{-5} m^2)

$$\text{Salt rejection (\%)} = \left(1 - \frac{C_P}{C_F}\right) \times 100\%$$

Table S2. Water flux and salt rejection calculation

**Membrane**	**Final conductivity**	**Water collected (L)**	**Swept liquid salt transfer**	**Initial salt (ppm), C_F**	**Salt rejection (%)**	**Water flux (L/m^2·h)**

			(ppm), C_P			
Pristine Al_2O_3	15.79 mS/cm	0.17888	26938.45	39292.85	31.44	240.94
UiO-66	13.83 mS/cm	0.16987	24761.58	38364.28	35.45	228.80
S1	6.81 mS/cm	0.00197	358.81	37364.28	99.03	2.65
S2	8.4 μS/cm	0.00417	242.27	39721.42	99.39	5.61
S3	9.4 μS/cm	0.00581	206.73	41292.85	99.49	7.82
S4	4.6 μS/cm	0.0111	25.68	39435.71	99.93	14.95
S5	24.6 μS/cm	0.00814	503.91	37078.57	98.64	10.96
U1	20.7 mS/cm	0.00172	1952.11	39292.85	95.03	2.31
U2	37.9 mS/cm	0.0036	1597.43	39792.85	95.98	4.84
U3	14.6 mS/cm	0.0043	510.11	40578.57	98.74	5.79
U4	28.9 mS/cm	0.00644	764.36	39650	98.07	8.67
U5	27.11 mS/cm	0.0074	619.04	38292.85	98.38	9.96

.
